# Structures of *Plasmodium vivax* serine hydroxymethyltransferase: implications for ligand-binding specificity and functional control

**DOI:** 10.1107/S1399004714023128

**Published:** 2014-11-22

**Authors:** Penchit Chitnumsub, Aritsara Jaruwat, Pinpunya Riangrungroj, Wanwipa Ittarat, Krittikar Noytanom, Worrapoj Oonanant, Jarunee Vanichthanankul, Phimonphan Chuankhayan, Somchart Maenpuen, Chun-Jung Chen, Pimchai Chaiyen, Yongyuth Yuthavong, Ubolsree Leartsakulpanich

**Affiliations:** a National Center for Genetic Engineering and Biotechnology (BIOTEC), National Science and Technology Development Agency (NSTDA), 113 Thailand Science Park, Phahonyothin Road, Klong Nueng, Klong Luang, Pathum Thani 12120, Thailand; b Life Science Group, Scientific Research Division, National Synchrotron Radiation Research Center, Hsinchu 30076, Taiwan; c Department of Biochemistry, Faculty of Science, Burapha University, Chonburi 20131, Thailand; d Department of Biochemistry and Center for Excellence in Protein Structure and Function, Faculty of Science, Mahidol University, Bangkok 10400, Thailand

**Keywords:** *Plasmodium vivax*, serine hydroxymethyltransferase, antimalarial targets, d-serine, (6*R*)-5-formyltetrahydrofolate, redox switch

## Abstract

*Plasmodium* parasites, the causative agent of malaria, rely heavily on *de novo* folate biosynthesis, and the enzymes in this pathway have therefore been explored extensively for antimalarial development. Serine hydroxymethyltransferase (SHMT) from *Plasmodium* spp., an enzyme involved in folate recycling and dTMP synthesis, has been shown to catalyze the conversion of l- and d-serine to glycine (Gly) in a THF-dependent reaction, the mechanism of which is not yet fully understood. Here, the crystal structures of *P. vivax* SHMT (*Pv*SHMT) in a binary complex with l-serine and in a ternary complex with d-serine (d-Ser) and (6*R*)-5-formyl­tetra­hydro­folate (5FTHF) provide clues to the mechanism underlying the control of enzyme activity. 5FTHF in the ternary-complex structure was found in the 6*R* form, thus differing from the previously reported structures of SHMT–Gly–(6*S*)-5FTHF from other organisms. This suggested that the presence of d-Ser in the active site can alter the folate-binding specificity. Investigation of binding in the presence of d-Ser and the (6*R*)- or (6*S*)-5FTHF enantiomers indicated that both forms of 5FTHF can bind to the enzyme but that only (6*S*)-5FTHF gives rise to a quinonoid intermediate. Likewise, a large surface area with a highly positively charged electrostatic potential surrounding the *Pv*SHMT folate pocket suggested a preference for a polyglutamated folate substrate similar to the mammalian SHMTs. Furthermore, as in *P. falciparum* SHMT, a redox switch created from a cysteine pair (Cys125–Cys364) was observed. Overall, these results assert the importance of features such as stereoselectivity and redox status for control of the activity and specificity of *Pv*SHMT.

## Abstract 2

Crystal structures of *P. vivax* serine hydroxymethyltransferase (*Pv*SHMT) in complex with l-serine and with d-serine and 5-formyltetrahydrofolate provide better understanding of ligand binding and the catalytic mechanism. Features that are important for controlling the activity and specificity of *Pv*SHMT such as stereoselectivity and redox status are addressed.

## 1. Introduction   

Vivax malaria is prevalent in many tropical and subtropical regions. In contrast to *Plasmodium falciparum* (*Pf*), which causes virulent malaria and has been studied extensively, *P. vivax* (*Pv*) has received less attention and is considered to be a neglected pathogen since it usually causes benign symptoms. However, *Pv* infection leads to serious complications such as severe anaemia, malnutrition, spleen enlargement and coma (Quispe *et al.*, 2014 ▶; Tan *et al.*, 2008 ▶; Kochar *et al.*, 2005 ▶; Beg *et al.*, 2002 ▶). Similar to falciparum malaria, drug resistance in vivax malaria is widespread (Tjitra *et al.*, 2008 ▶). Therefore, the disease threat owing to *Pv* infection should not be underestimated.

*Plasmodium* serine hydroxymethyltransferase (SHMT) has recently been shown to be vital for parasite growth and development, making it a potential target for antimalarial drug development (Pornthanakasem *et al.*, 2012 ▶). SHMT is a pyridoxal-5′-phosphate (PLP)-dependent enzyme that catalyzes the interconversion of l-serine (l-Ser) and tetrahydro­folate (THF) to glycine (Gly) and methylene tetrahydrofolate (MTHF). The enzyme provides activated one-carbon units for the synthesis of dTMP, choline and amino acids (Müller & Hyde, 2013 ▶). Heterologously expressed *Pv*SHMT was found to exhibit both THF-dependent and THF-independent catalytic activities, and could use d-serine in the THF-dependent reaction. However, unlike the binding of l-Ser, an enzyme–quinonoid intermediate was only observed upon binding with d-Ser (Sopitthummakhun *et al.*, 2009 ▶).

At present, structural coordinates for SHMT are available from nine organisms. Most of them are internal aldimines with a catalytic lysine residue linked to PLP or external aldimines with PLP linked to l-Ser or Gly. Among the *Bacillus stearo­thermophilus* SHMT (*bs*SHMT) structures, one is a PLP–l-*allo*-threonine (PLP-l-alloThr) Schiff base, while the others are PLP–Gly Schiff bases despite the presence of PLP and l-*allo*-Thr during crystallization (Pai *et al.*, 2009 ▶). Three structures of PLP–Gly and 5-formyltetrahydrofolate (5FTHF) complexes of *bs*SHMT, *E. coli* SHMT (*ec*SHMT) and mammalian mouse cytosolic SHMT (*m*SHMT) are also available (Trivedi *et al.*, 2002 ▶; Scarsdale *et al.*, 2000 ▶; Szebenyi *et al.*, 2000 ▶). In the tetrameric structure of rabbit cytosolic SHMT (*r*SHMT), triglutamated 5FTHF (triGlu-5FTHF) with a PLP internal aldimine was found in two active sites, while a PLP–Gly complex was found in the other two active sites (Fu *et al.*, 2003 ▶), and interestingly it also indicates favourable binding of the poly-γ-glutamate side chain of THF to mammalian SHMTs. Recently, the structure of *Pf*SHMT with a PLP internal aldimine revealed a cysteine pair at the THF pocket entrance acting as a redox switch that regulates the THF-dependent activity of SHMT, a unique feature of *Plasmodium* SHMT (Chitnumsub *et al.*, 2014 ▶). The distinct biochemical properties of *Plasmodium* SHMT and human SHMT (*h*SHMT; Pinthong *et al.*, 2014 ▶) can be used to design therapeutic species-specific inhibitors.

Although the structures of various complexes are essential for new antimalarial drug design and development, only one *Plasmodium* SHMT structure, that of *Pf*, has been successfully determined to date. In the present study, we have determined the structures of the binary complex *Pv*SHMT–l-Ser and the ternary complex of *Pv*SHMT with d-Ser and 5FTHF at 2.5 and 2.4 Å resolution, respectively. Differences in the accommodation of l-Ser and d-Ser in the *Pv*SHMT active site may account for the broad substrate specificity of SHMT and the lower catalytic efficiency for d-Ser in the THF-dependent catalytic function. In addition, the binding of d-Ser promoted the binding of (6*R*)-5FTHF instead of (6*S*)-5FTHF. Finally, a redox switch similar to that in *Pf*SHMT which can be induced by an oxidant drug such as methylene blue was found based on reversible disulfide formation between two cysteine residues.

## 2. Materials and methods   

### 2.1. Expression and purification of *Pv*SHMT   

*E. coli* BL21 (DE3) cells harbouring the pET-17b-*Pv*SHMT plasmid were cultured in Luria–Bertani medium containing 100 µg ml^−1^ ampicillin at 37°C. Expression of the *Pv*SHMT protein was induced by 0.4 m*M* IPTG when the culture OD_600_ reached 1 and was followed by incubation at 20°C for 22 h. The cells were harvested by centrifugation at 4420*g* for 10 min and were stored at −20°C. The frozen cell paste from a 6 l culture was resuspended in lysis buffer [25 m*M* HEPES pH 8.3, 0.5 m*M* EDTA, 1 m*M* dithiothreitol (DTT), 20%(*v*/*v*) glycerol] and passed though a French press at 10.3 MPa on ice three times. Soluble protein was fractionated by centrifugation at 39 000*g* for 1 h, and 20 µ*M* PLP was added to the crude extract followed by incubation with gentle stirring on ice for 30 min to prevent cofactor loss and to ensure formation of the holoenzyme. *Pv*SHMT was then fractionated by 0–30 and 30–80%(*w*/*v*) ammonium sulfate precipitation. After centrifugation at 39 000*g* for 30 min at 4°C, the 30–80%(*w*/*v*) ammonium sulfate precipitated protein fraction was resuspended and then dialyzed twice against 2 l lysis buffer at 4°C using a 10 kDa molecular weight cutoff dialysis bag. The dialyzed protein was centrifuged at 39 000*g* for 30 min at 4°C and applied onto a DEAE-Sepharose column (2.5 × 75 cm). The column was washed with two column volumes of lysis buffer plus 5 m*M* NaCl. *Pv*SHMT was eluted stepwise using two column volumes each of lysis buffer plus 10, 15 and 20 m*M* NaCl. The *Pv*SHMT fractions were yellow with an absorption peak at 422 nm. Fractions showing a single band of molecular weight 49 kDa on 12% SDS–PAGE were pooled and concentrated using Millipore centrifugal concentrators with 30 kDa molecular-weight cutoff. Purified *Pv*SHMT with freshly added 10 µ*M* PLP was exchanged into 25 m*M* HEPES pH 7.0, 0.5 m*M* EDTA, 10 m*M* DTT, 20%(*v*/*v*) glycerol. The protein concentration was assessed using the technique of Bradford (1976 ▶) using BSA as a standard. The purified *Pv*SHMT was stored at −80°C.

### 2.2. Interaction of *Pv*SHMT with d-Ser in the presence of (6*R*)- and (6*S*)-5FTHF and enzyme inhibition by (6*R*)- and (6*S*)-5FTHF   

In order to observe enzyme spectrum changes in the presence of d-Ser and pure enantiomers of 5FTHF [(6*S*) or (6*R*); Merck Epova AG, Schaffhausen, Switzerland], *Pv*SHMT (30 µ*M*; *A*_422_ = 0.2) was incubated with folate (7.9 µ*M*) and the spectrum was recorded using a diode-array spectrophoto­meter. d-Ser (50 µ*M*) was then added and the spectral change was monitored for 20 min. The study was performed in 50 m*M* HEPES pH 8.0, 0.5 m*M* EDTA, 1 m*M* DTT at 25°C.

*Pv*SHMT activity was determined using a *Pv*SHMT–methylene tetrahydrofolate dehydrogenase (MTHFD) coupling assay as described previously (Sopitthummakhun *et al.*, 2012 ▶). Inhibition by either (6*S*)- or (6*R*)-5FTHF was assessed by measuring the initial velocity of the reaction in the presence of each enantiomer of 5FTHF [0.05–1.88 m*M* for (6*S*)-5FTHF and 1.55 m*M* for (6*R*)-5FTHF]. The inhibition constant (*K*_i_) was calculated for (6*S*)-5FTHF based on the equation *V* = (*V*_max_ *K*_i_)/(*K*_i_ + [I]) and the percentage inhibition was used to represent the inhibition efficacy of (6*R*)-5FTHF.

### 2.3. Identification of a redox switch   

To determine whether *Pv*SHMT activity is affected by the redox environment, *Pv*SHMT (100 µ*M*) prepared in the absence of DTT was incubated with 10 m*M* DTT, and an aliquot of the enzyme (0.5 µ*M*) was taken for initial velocity measurements at different time points. To test activity under oxidizing conditions, a similar experiment was performed by incubating the protein prepared in the presence of DTT with 50 m*M* hydrogen peroxide (H_2_O_2_). The percentage enzyme activity in the presence of DTT or H_2_O_2_ was plotted against incubation time.

Redox switching was also monitored by incubating the enzyme (100 µ*M*) in the presence of DTT (10 m*M*) followed by H_2_O_2_ (50 m*M*) or methylene blue (MB; 1 m*M*) and then DTT (10 m*M*). Activity was measured at different time points.

### 2.4. Crystallization of *Pv*SHMT   

*Pv*SHMT was crystallized using the microbatch method in a 60-well plate (1 mm diameter at the bottom of each well) covered with 6 ml baby oil (a mixture of mineral oil, olive oil and vitamin E; PZ Cussons, Thailand; Chitnumsub *et al.*, 2004 ▶). Protein–ligand complexes were prepared by mixing 0.38 m*M* *Pv*SHMT with 0.86 m*M* PLP, 62 m*M* β-mercaptoethanol (BME), 87 m*M* Gly, l-Ser or d-Ser and 43 m*M* 5FTHF (Sigma–Aldrich, USA). The protein mixture was equilibrated on ice for 30 min for complex formation. Crystallization was set up on a microplate by first pipetting 1 µl crystallization solution into a well, which was then layered with 6 ml oil followed by addition of 1 µl of the protein complex through the oil layer. Protein crystals of *Pv*SHMT grew to optimum size in 1–4 d at 293 K in 20–21%(*w*/*v*) PEG 4000, 70–90 m*M* NaCl, 100 m*M* Tris–HCl pH 8.5, 15%(*v*/*v*) trifluoroethanol (TFE).

### 2.5. Structure determination   

A single crystal was flash-vitrified in liquid nitrogen using 20%(*v*/*v*) glycerol in the crystallization condition as a cryo­protectant. X-ray diffraction data were collected at 100 K at a wavelength of 1 Å using an ADSC Quantum 315r CCD detector on beamline 13B1 at NSRRC, Taiwan. Data were processed using the *HKL*-2000 package (Otwinowski & Minor, 1997 ▶). X-ray diffraction data and refinement statistics are listed in Table 1 ▶. The structure of *Pv*SHMT was determined by molecular replacement using *Phaser* in the *CCP*4 suite (Winn *et al.*, 2011 ▶) with the chain *A* protomer from the *Pf*SHMT coordinates (PDB entry 4o6z; 83% identical to *Pv*SHMT) as the phasing model (Chitnumsub *et al.*, 2014 ▶). Model building and structure refinement were carried out using *Coot* (Emsley & Cowtan, 2004 ▶; Emsley *et al.*, 2010 ▶) and *REFMAC*5 (Murshudov *et al.*, 2011 ▶), respectively. The structure was validated by *PROCHECK* (Laskowski *et al.*, 1993 ▶) and using the wwPDB validation server. Superposition of structures was carried out using *LSQMAN* (Kleywegt, 1996 ▶) and figures were prepared using the *PyMOL* molecular-graphics program (http://www.pymol.org).

### 2.6. PDB codes   

Atomic coordinates and structure factors have been deposited with the following PDB codes: 4pff for the PLP internal aldimine, 4pfn for the PLP–l-Ser external aldimine and 4oyt for the PLP–d-Ser aldimine with (6*R*)-5FTHF.

## 3. Results and discussion   

### 3.1. Crystallization   

The *Pv*SHMT complexes crystallized in two different space groups: *P*3_2_ and *C*2. Space group *P*3_2_ with unit-cell parameters *a* = 58.30, *b* = 58.30, *c* = 473.31 Å, α = β = 90, γ = 120° was obtained for the complex with an amino-acid substrate or mimic without folate, while space group *C*2 with unit-cell parameters *a* = 100, *b* = 58, c = 237 Å, α = γ = 90, β = 90.01° was obtained in the presence of a folate derivative. Although the diffraction quality of the crystals in the two space groups was comparable, a lower resolution was obtained for the *P*3_2_ crystal data owing to incompleteness of the high-resolution shell caused by overlapping spots along the longest axis, and these data were not used for further analysis. Two crystals of particular interest were obtained in space group *C*2. The first was a crystal of the binary complex of *Pv*SHMT with l-Ser, which diffracted to 2.5 Å resolution and was obtained in the presence of methotrexate (MTX), although there was no density for MTX in the final map. The second was a ternary complex of *Pv*SHMT with d-Ser and 5FTHF, which included positive density for d-Ser and (6*R*)-5FTHF (2.4 Å resolution).

### 3.2. Crystal structure of *Pv*SHMT   

The overall structure of *Pv*SHMT was found to be very similar to that of *Pf*SHMT (Chitnumsub *et al.*, 2014 ▶), existing as a twofold symmetric homodimer with two catalytic sites. The folate binding pocket of *Pv*SHMT is distinct from that of bacterial SHMTs but is similar to that of mammalian SHMTs, although the kinetic properties of *Pv*SHMT and mammalian SHMT reflect the subtle differences in the binding pockets and overall quaternary structures (Pinthong *et al.*, 2014 ▶). The crystal structure of *Pv*SHMT with d-Ser and 5FTHF shows that the folate pocket of *Pv*SHMT is 1 Å wider than those of mammalian and bacterial SHMTs, possibly affecting the interactions and affinity between the active-site residues of *Pv*SHMT and folate analogues (see §[Sec sec3.4]3.4).

**Figure 1:**
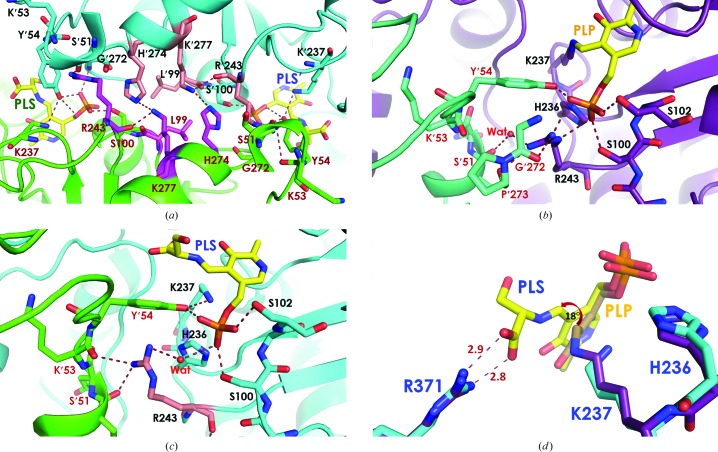
Crystal structure of *Pv*SHMT. (*a*) The dimer interface of two PLP and amino-acid substrate pockets with interactions contributed by Leu99, Arg243, His274 and Lys277 from protomers *A* (coloured cyan and pink) and *B* (coloured green and magenta). (*b*, *c*) Conformation of Arg243 (*b*) in the PLP–Lys237 Schiff-base complex and (*c*) in the PLP–l-Ser Schiff-base complex (PLS). (*d*) Superposition of the 4pff (violet) and 4pfn (cyan) dimers shows the rotation of the PLP ring by 18° from the PLP–Lys237 Schiff base (orange) to the PLP–l-Ser Schiff base (yellow).

On the other hand, the PLP and amino-acid pockets of SHMTs from all organisms appear to be alike. The two active sites for the PLP cofactor in the *Pv*SHMT homodimer are very close, with distances between PLP α-phosphate moieties of the two active sites of 18.2 Å and distances between C4A (an aldehyde C atom) of 27 Å (Fig. 1 ▶ *a*). The interface of the two protomers separating the active site comprises both hydrophobic and electrostatic interactions owing to residues Leu99, Arg243, His274 and Lys277 (Fig. 1 ▶ *a*). Electrostatic interactions include hydrogen bonds from the His274 side chain to the Lys277 side chain and the Arg243 backbone of the second protomer and from the Ser51 carbonyl backbone to the Arg243 side chain. Starting from Lys237 in the middle of the active site, the PLP amino-acid pocket is surrounded by five conserved loops including residues 99–102, 181–185 and 234–237 from the first protomer and 51–64 and 272–274 from the second protomer. Residues Tyr′54, Glu′56 and Tyr′64 in the 51–64 or ‘YEY’ (SNK**Y**S**E**GYPKKRY**Y**) loop are important for substrate binding and catalysis (Szebenyi *et al.*, 2004 ▶; Rajaram *et al.*, 2007 ▶), with Glu′56 and Tyr′64 being particularly flexible (see §§[Sec sec3.3]3.3 and [Sec sec3.4]3.4).

In structures with and without amino-acid substrates, the hydrogen-bond network at the PLP phosphate moiety is significantly different at Arg243, which adopts different side-chain conformations (Figs. 1 ▶ *b* and 1 ▶ *c*). In the internal aldimine complex, the Arg243 side chain hydrogen bonds directly to the PLP phosphate group and to the main-chain carbonyl of Gly′272  and Pro′273 (Fig. 1 ▶ *b*). In contrast, in the external aldimine form of PLP bound to l-Ser the Arg243 side chain does not interact with PLP directly, but rather *via* a water bridge to the PLP phosphate moiety and direct hydrogen bonding to the main-chain carbonyl of Ser′51 on the 3_10_-helix and Lys′53 of the YEY loop (Fig. 1 ▶ *c*). This interaction with Ser′51 and Lys′53 probably provides additional anchoring interactions for holding the YEY loop in place during THF-dependent reactions. Overall, the difference in Arg243 conformation between the two PLP complexes is suggestive of functional significance of Arg243 in *Pv*SHMT catalysis. Finally, the internal and external aldimine forms also exhibit conformational differences in the PLP pyridoxal ring (Fig. 1 ▶ *d*), which is rotated by 18° at C4A with a focal point at the pyridine N atom in the PLP Schiff base.

### 3.3. Binding of d-Ser and l-Ser   

The structures of the *Pv*SHMT–l-Ser and *Pv*SHMT–d-Ser–5FTHF complexes were used to assess the amino-acid binding pocket. Like the l-Ser substrate, d-Ser was found in the crystal structure as an external aldimine with PLP, indicating that d-Ser is similarly capable of replacing the PLP–Lys237 Schiff base. This result agrees with the biochemical characterization of SHMT, which showed that the enzyme is catalytically active towards both serine isomers (Sopitthummakhun *et al.*, 2009 ▶).

**Figure 2:**
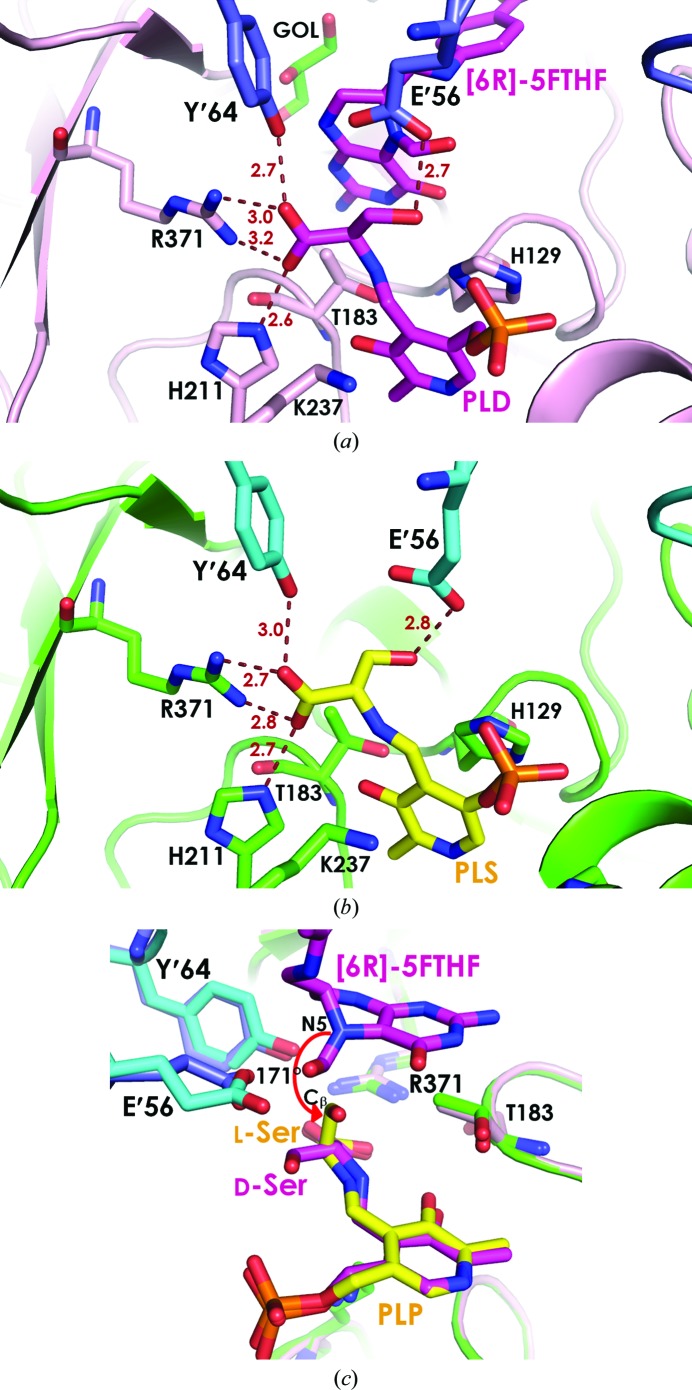
Amino-acid binding pocket of *Pv*SHMT. (*a*) d-Ser in the ternary complex *Pv*SHMT–d-Ser–5FTHF with the PLP–d-Ser (PLD) Schiff base and (6*R*)-5FTHF coloured in magenta in protomer *A* (pink) and protomer *B* (blue) with a glycerol (GOL) molecule in green. (*b*) l-Ser in the binary complex *Pv*SHMT–l-Ser with a PLP–l-Ser (PLS) Schiff base. (*c*) Superposition of the two complexes showing the configurations of d-­Ser and l-Ser.

Key interactions with both d-Ser and l-Ser consist of two components: hydrogen bonds from the conserved residues in the PLP pocket (Ser100, Ser102, Thr183, Asp208, His129, His236, Tyr′54 and Gly′272) and the amino-acid pocket (Ser34, His211, Arg371, Glu′56 and Tyr′64). Although the stabilizing interactions are from identical residues, three major differences between d-Ser and l-Ser binding can be observed. Firstly, the salt-bridge interactions between the Arg371 side chain and the carboxyl group of d-Ser are distorted, at distances of 3.0 and 3.2 Å, while two ideal salt bridges at distances of 2.7 and 2.8 Å are observed to l-Ser (Fig. 2 ▶). Also, d-Ser is mainly restrained by the side-chain carboxyl group of Glu′56 from a neighbouring protomer, with the d-Ser β-hydroxymethyl side chain binding in the space between the PLP phosphate moiety, Tyr′64 and Glu′56, and being sterically controlled by the carboxyl moiety of Glu′56 (Fig. 2 ▶ *a*). In contrast, the l-Ser binding pocket is extended towards the folate pocket and is surrounded by His129, Glu′56 and Tyr′64 (Fig. 2 ▶ *b*). Furthermore, superposition of the two complexes reveals different orientations of the d-Ser and l-Ser side chains (Fig. 2 ▶ *c*). The d-Ser side chain is oriented away from N5 of THF and is found in the extended plane of the PLP ring, while the l-Ser side chain is perpendicular to N5. Altogether, these differences may contribute the differences in *K*_m_ between d-Ser and l-Ser (47 ± 6 and 0.18 ± 0.03 m*M*, respectively; Sopitthummakhun *et al.*, 2009 ▶) and the overall catalytic efficiency (0.006 and 28 s^−1^ m*M* ^−1^, respectively). Nevertheless, these observations indicate that the overall amino-acid binding site of *Pv*SHMT is sufficiently plastic to accommodate both the d- and l-isomers of the substrate.

### 3.4. Binding of (6*R*)-5FTHF in the presence of d-Ser   

Folate binding can also be examined in the context of the *Pv*SHMT–d-Ser–5FTHF complex. Only (6*R*)-5FTHF was observed in the folate-binding pocket despite the use of a racemic mixture of 5FTHF for crystallization, suggesting that only the (6*R*)-5FTHF enantiomer binds to *Pv*SHMT–d-Ser or that only the (6*R*)-5FTHF complex crystallized. The former is less likely, as (6*S*)-5FTHF has been observed with a Gly external aldimine in SHMT structures from other organisms (Scarsdale *et al.*, 2000 ▶; Trivedi *et al.*, 2002 ▶; Fu *et al.*, 2003 ▶) and (6*S*)-THF is an active substrate with both l-Ser and d-Ser for *Pf*SHMT and *Pv*SHMT. (6*R*)-5FTHF binding is stabilized by van der Waals interactions from Tyr′63, Phe′266, Pro′267, Phe134, Leu130, Leu124 and Val141. In the three protomers in each asymmetric unit, the distances between the d-Ser β-hydroxyl group and the 5FTHF 5-formyl group are 2.6, 2.8 and 3.6 Å, indicating hydrogen-bonding and van der Waals interactions. Structural comparisons of the THF pocket show that the *Pv*SHMT pocket is 1 Å wider from His129 to Asn356, where the pterin ring of THF is located, compared with mammalian and bacterial SHMTs at the equivalent pair of residues (His126^*r*,*ec*^ to Asn347^*r*,*ec*^).

**Figure 3:**
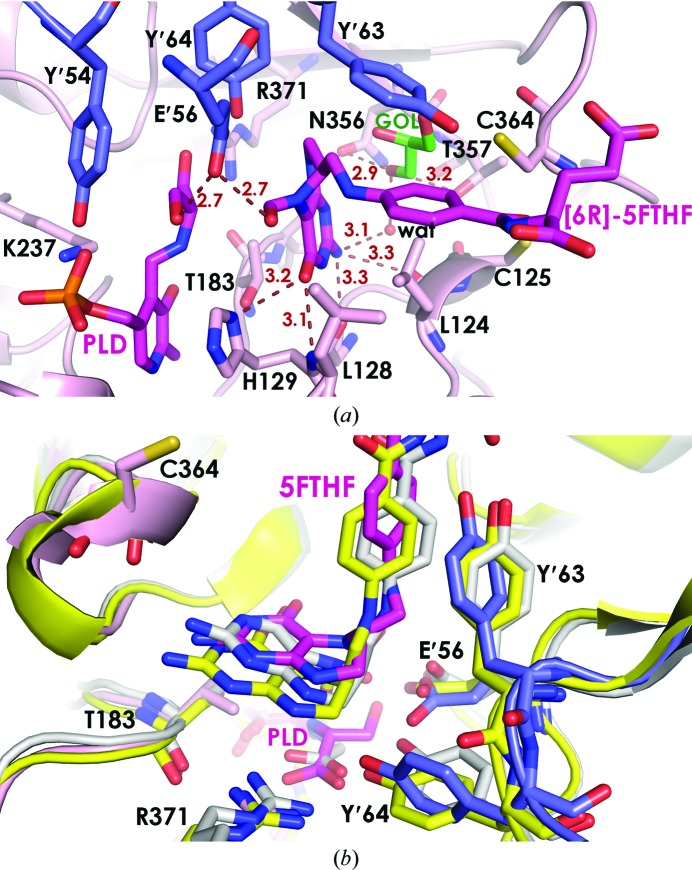
Binding of (6*R*)-5FTHF in the ternary *Pv*SHMT–d-Ser–(6*R*)-5FTHF complex. (*a*) Interactions between *Pv*SHMT and (6*R*)-5FTHF, with dashed lines indicating hydrogen-bond distances in Å from residues in protomers *A* (pink) and *B* (blue) to the substrates PLP–d-Ser Schiff base (PLD) and (6*R*)-5FTHF in magenta and a glycerol (GOL) molecule in green. A water molecule (wat; red sphere) forms a bridge between 5FTHF and the protein. (*b*) Superposition of *Pv*SHMT–d-Ser–(6*R*)-5FTHF (in blue and pink), *ec*SHMT–Gly–(6*S*)-5FTHF (PDB entry 1dfo, white) and *r*SHMT–PLP–(6*S*)-triGlu-5FTHF (PDB entry 1ls3, yellow). Residue labelling is based on the *Pv*SHMT sequence.

Interestingly, this wider pocket may have allowed the accommodation of a glycerol molecule in one active pocket of each native enzyme, occupying the space between (6*R*)-5FTHF and residues 354–357 and providing additional interaction between the protein and 5FTHF (Fig. 3 ▶ *a*). The first hydroxyl group of the glycerol forms a hydrogen bond to the main-chain carbonyl of Lys355 (2.9 Å), and the second hydroxyl group forms a hydrogen bond to the side chains of Asn356 (2.9 Å) and Thr357 (3.2 Å). A water molecule links the second hydroxyl group (2.6 Å) and the 2-NH_2_ moiety (3.1 Å) of the (6*R*)-5FTHF pterin ring. The third hydroxyl group of the glycerol was 3.4 Å from the sulfhydryl group of Cys364, which is one of a pair of cysteines that are involved in forming the redox switch that was found to regulate *Pf*SHMT activity (Chitnumsub *et al.*, 2014 ▶). These observations indicate interactions that can be useful for inhibitor design. In contrast, the binding of (6*S*)-5FTHF in *r*SHMT, *ec*SHMT and *bs*SHMT is stabilized *via* direct hydrogen bonding to Asn347^*r*,*ec*^ and Asn341^*bs*^, which are equivalent to Asn356*^Pv^*.

The position of the bound (6*R*)-5FTHF is also subtly different from that of (6*S*)-5FTHF in other SHMTs (Fig. 3 ▶ *b*). Amino-acid sequence alignment suggests that THF binding to *Pv*SHMT may be influenced by steric hindrance from the nonconserved Thr183*^Pv^* (equivalent to Ser203*^h^* and Ser175^*r*,*ec*^), lying underneath the pteridine ring of 5FTHF or THF. The distance of the β-methyl group of Thr183*^Pv^* to C4a or C8a of the 5FTHF pterin ring in *Pv*SHMT–d-Ser–5FTHF is 3.9–4.1 Å, while that of the C^β^ atom of Ser175*^r^* to the same atoms of 5FTHF is 4.1–4.4 Å in *r*SHMT, suggesting a shift in THF positioning. *Pv*SHMT binding of (6*R*)-5FTHF and the d-Ser external aldimine alters the conformation of at least three residues, namely Glu′56, Tyr′63 and Tyr′64 (Fig. 3 ▶ *b*).

### 3.5. Probing the interactions of 5FTHF in the presence of serine isomers   

**Figure 4:**
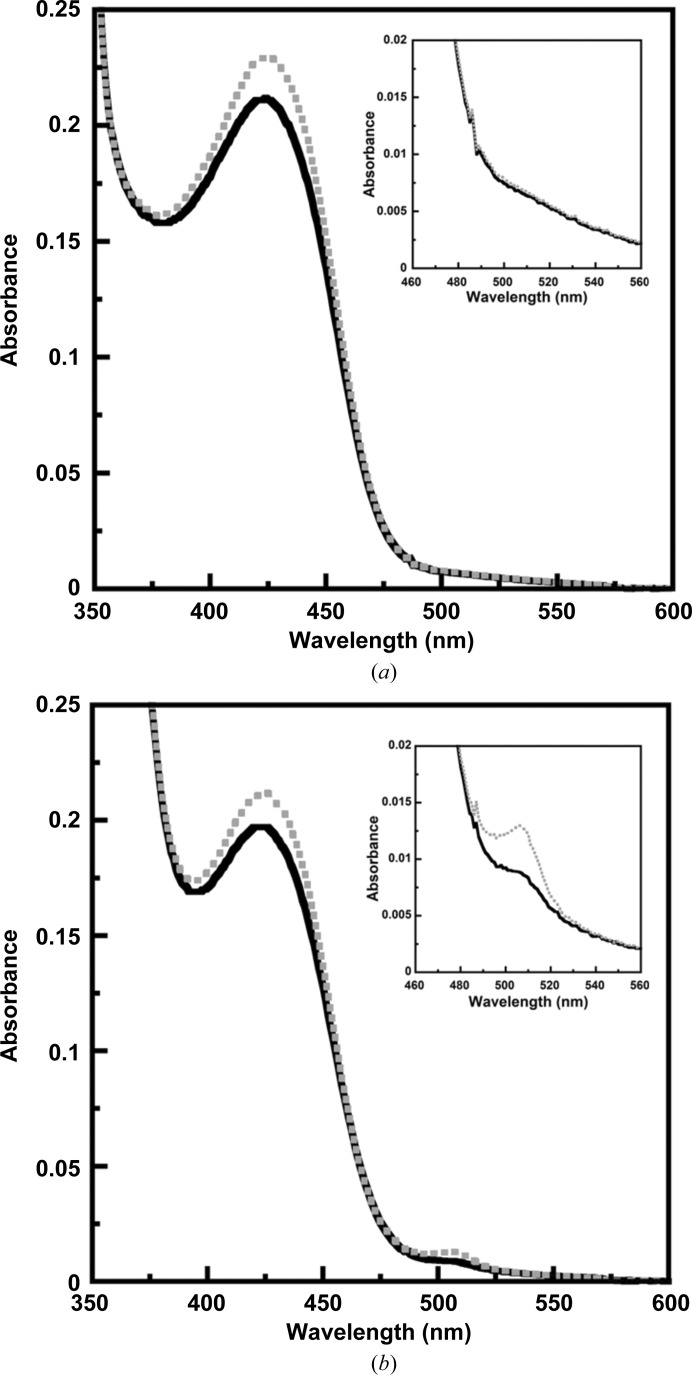
Spectral change of *Pv*SHMT upon binding of d-Ser in the presence of (*a*) (6*R*)-5FTHF and (*b*) (6*S*)-5FTHF. The solid line is the spectrum of *Pv*SHMT in the presence of 5FTHF, while the dashed line is the spectrum after the addition of d-Ser.

To verify external aldimine formation with d-Ser in the presence of (6*R*)-5FTHF in solution, spectroscopic studies of *Pv*SHMT with d-Ser and either (6*S*)- or (6*R*)-5FTHF were performed. The formation of an external aldimine (420 nm) was observed for the binding of d-Ser to *Pv*SHMT in the presence of (6*R*)-5FTHF (Fig. 4 ▶ *a*), while an additional peak at 500 nm indicative of an enzyme–quinonoid intermediate was detected for (6*S*)-5FTHF (Fig. 4 ▶ *b*). These suggested that the stereoisomer of 5FTHF has an effect on the binding of d-Ser to *Pv*SHMT, resulting in different intermediates.

The interaction of *Pv*SHMT with each enantiomer of 5FTHF was further explored based on an inhibition study using an SHMT–MTHFD coupling assay *via* l-Ser–THF. Inhibition *via* d-Ser–THF was not studied owing to the low catalytic efficiency of the d-Ser substrate. Interestingly, while (6*S*)-5FTHF inhibited *Pv*SHMT with a *K*_i_ of 0.21 m*M*, (6*R*)-5FTHF inhibited *Pv*SHMT only slightly, with the activity decreasing by 30% at 1.55 m*M*. The results suggest that the binding affinity of (6*S*)-5FTHF to *Pv*SHMT in the presence of l-Ser is higher than that of (6*R*)-5FTHF.

### 3.6. Proposed *Pv*SHMT catalytic mechanism for the THF-dependent reaction   

The mechanism of the THF-dependent SHMT reaction is still unclear. The reaction either proceeds through retro-aldol cleavage to form free formaldehyde before the formation of MTHF, or the N5 atom of THF directly participates in nucleophilic replacement to form Gly (Schirch & Szebenyi, 2005 ▶; Schirch, 1998 ▶; Trivedi *et al.*, 2002 ▶). The likelihood of nucleophilic replacement was examined by superposition of *bs*SHMT co-complexed with (6*S*)-5FTHF and PLP–Gly (PDB entry 1kl2), PLP–Gly (PDB entry 1kl1) or PLP–l-Ser adducts (PDB entry 1kkp), revealing a sufficiently close distance of 2.8 Å between N5 of 5FTHF and C^β^ of the l-Ser external aldimine but an angle of 150° from the pteridine ring to the C^β^ methylene group of the l-Ser external aldimine. Nucleophilic displacement would require the serine C^β^ methylene group to be perpendicular to the pteridine ring of THF or to align close to 180° with the *p* orbital of N5. Unfortunately, PLP–l-Ser adducts of *m*SHMT and *r*SHMT are not available for additional analysis (Szebenyi *et al.*, 2000 ▶; Fu *et al.*, 2003 ▶). The *Pv*SHMT structures were therefore used to probe the position of THF and to gain insights into the SHMT mechanism.

Structures of *Pv*SHMT–l-Ser and *Pv*SHMT–d-Ser–(6*R*)-5FTHF were superimposed to pinpoint the position of THF in the pocket (Fig. 2 ▶ *c*). The N5 atom of 5FTHF was found to be perpendicular to the serine C^β^ methylene group and within 3 Å, which is shorter than the van der Waals distance, rendering nucleophilic attack feasible. Moreover, the C^α^—C^β^—N5 angle is 171°, which is nearly a 180° alignment with the *p* orbital of N5. Therefore, these structures support the proposal that *Pv*SHMT undergoes nucleophilic displacement in catalyzing the reaction between l-Ser and THF in the crystallization conditions at pH 8.

### 3.7. Implication for the preferred accommodation of poly-γ-glutamate–THF   

Two solvent-exposed loops on the periphery of the folate-binding pocket were examined to assess whether *Pv*SHMT has a preference for polyglutamated or monoglutamated THF. In the *Pv*SHMT–d-Ser–5FTHF structure, the two stretches (residues Asp136–Lys140 of loop-C125*^Pv^* and Asp361–Ser366 of loop-C364*^Pv^*) have the highest temperature factors, with an average *B* value of 70 Å^2^ compared with 44 Å^2^ for the overall structure, suggesting that even in the presence of bound (6*R*)-5FTHF there is flexibility similar to that in structures solved without bound folate (Chitnumsub *et al.*, 2014 ▶). The positively charged Lys138 and Lys139 side-chain amines of loop-C125*^Pv^* are 6.0–6.5 Å from the l-glutamate group of monoglutamated (6*R*)-5FTHF and unexpectedly remain in the same conformation in the presence or absence of bound folate, similar to their equivalent in *r*SHMT complexed with triGlu-5FTHF and PLP. This *r*SHMT ternary-complex structure (PDB entry 1ls3; Fu *et al.*, 2003 ▶) shows a distance of 3.1 Å between the α-carboxyl of the third glutamate moiety and the Lys134 side-chain amine situated on the apex of its loop-C125 equivalent, compared with 7.5 Å for the α-carboxyl of the first Glu moiety of triGlu-5FTHF. Whether or not the unchanged conformation of the loop observed for *Pv*SHMT is owing to the preferred accommodation of polyglutamated THF substrates is unclear. However, unlike mammalian and *Plasmodium* SHMTs, bacterial SHMTs contain only a short loop of small hydrophobic side chains (*ec*SHMT-P132V133) in the corresponding position and only accommodate monoglutamated THF.

Loop-C364*^Pv^*, on the other hand, is adjacent to the *p*-aminobenzoic acid (*p*ABA) moiety of 5FTHF. Its equivalents in *ec*SHMT and *bs*SHMT adopt a closed conformation in the presence of bound 5FTHF, in which Pro356*^ec^* makes van der Waals contact with the *p*ABA moiety and Phe357*^ec^* makes a π-edge interaction with Tyr′64*^ec^* (equivalent to Tyr′63*^Pv^*). This conformational change in bacterial SHMTs contributes to the THF binding affinity. However, such a closed conformation was not observed in *Plasmodium* and mammalian SHMTs (Fu *et al.*, 2003 ▶).

**Figure 5:**
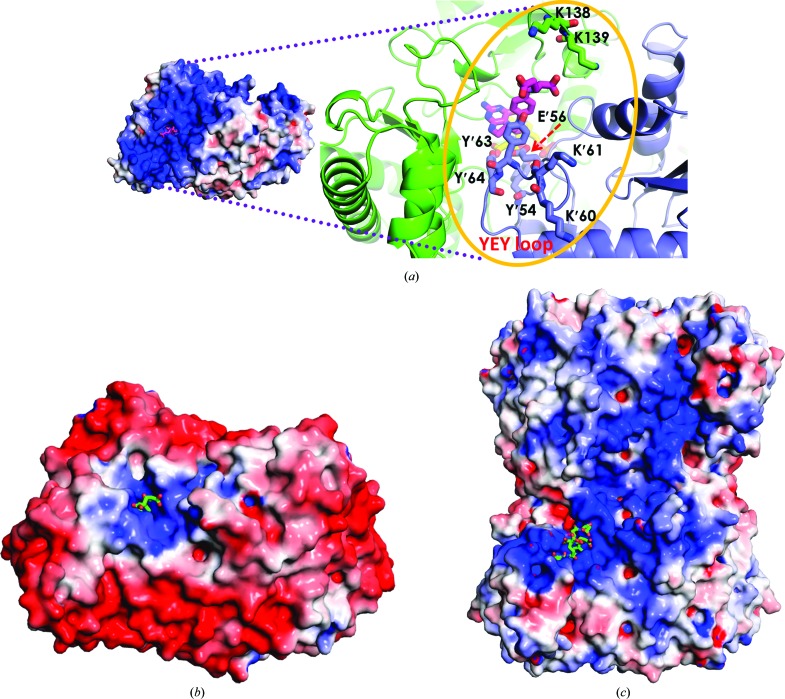
Surface electrostatic potential. (*a*) Dimeric *Pv*SHMT–d-Ser–(6*R*)-5FTHF, with the inset showing key residues involved in folate binding: Tyr′54, Glu′56, Lys′60, Lys′61, Tyr′63 and Tyr′64 in the YEY loop and Lys138 and Lys139 in loop-C125. (*b*) Dimeric *ec*SHMT (PDB entry 1dfo). (*c*) Tetrameric *r*SHMT (PDB entry 1ls3). Each structure contains 5FTHF, but that of *r*SHMT contains triGlu-5FTHF.

Another difference lies in the SHMT architecture and the electrostatic potential. The surface of mammalian and *Plasmodium* SHMTs is positively charged near the folate glutamate moiety, while that of bacterial SHMTs is negatively charged (Fig. 5 ▶). Additionally, positively charged residues, namely Lys138 and Lys139 on loop-C125*^Pv^* and Lys′60 and Lys′61 on the YEY loop of the second protomer, are located on the surface of the THF-binding pocket (Fig. 5 ▶ *a*). It is likely that these residues contribute to the binding affinity of poly-γ-­glutamate–THF.

Altogether, the positively charged surface and the characteristics of loop-C125*^Pv^* and loop-C364*^Pv^* suggest that *Plasmodium* SHMT may preferentially accommodate the poly-γ-­glutamate side chain of THF rather than the mono-γ-­glutamate side chain. This is in good agreement with previous findings that a polyglutamated THF pool is present in *Plasmodium* by the conversion of *p*ABA to THF with 3–5 glutamate residues (Wang *et al.*, 2004 ▶; Müller & Hyde, 2013 ▶).

### 3.8. Oxidative inhibition in *Pv*SHMT   

**Figure 6:**
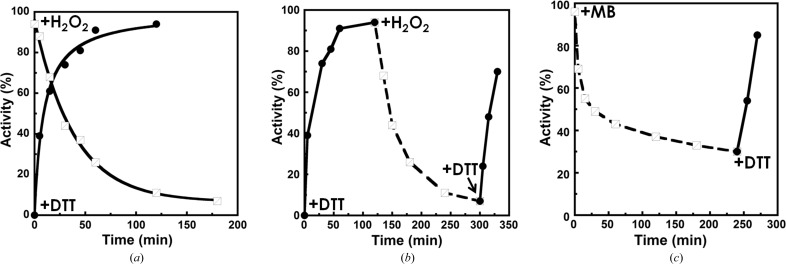
THF-dependent activity of *Pv*SHMT in relation to the redox environment. (*a*) Time course of *Pv*SHMT activity in the presence of DTT (black circles) and H_2_O_2_ (empty squares). (*b*, *c*) Alteration of *Pv*SHMT activity in response to redox switching using the oxidant–reductant pairs DTT–H_2_O_2_ and DTT–methylene blue (DTT, solid line with black circles; H_2_O_2_ and methylene blue, dashed line with empty squares), respectively.

A previous study revealed that the THF-dependent activity of *Pf*SHMT is regulated by the redox status of a cysteine pair (Chitnumsub *et al.*, 2014 ▶). Sequence conservation (Cys125 and Cys364) and the presence of the sulfhydryl form in the *Pv*SHMT–d-Ser–5FTHF and *Pv*SHMT–l-Ser complexes and the disulfide in the *Pv*SHMT–PLP structure prompted an examination of the dependence of *Pv*SHMT activity on its redox state. In this study, H_2_O_2_ and DTT were used as oxidizing and reducing agents, respectively. The presence of DTT (10 m*M*) led to ∼40% enzyme activity within 5 min of incubation at 0°C and to 90% activity within 1 h (Fig. 6 ▶ *a*). The addition of 50 m*M* H_2_O_2_ to *Pv*SHMT in 10 m*M* DTT resulted in a decreased *Pv*SHMT activity over time to less than 10% after a 3 h incubation (Fig. 6 ▶ *a*).

Switching of enzyme activity based on the redox environment was also observed for *Pv*SHMT. The activity was altered sequentially when it was incubated with DTT, H_2_O_2_ and then DTT (Fig. 6 ▶ *b*). Based on the results obtained, the sensitivity of the enzyme to the antimalarial drug methylene blue, a redox dye, was then examined. The effect was similar to that of H_2_O_2_ but at a slower rate, and was reversed if DTT was subsequently added (Fig. 6 ▶ *c*). These observations indicated that *Pv*SHMT activity can be controlled through a redox switch like that of *Pf*SHMT. The oxidation/reduction reactions were slow, suggesting that there are other steps occurring as a result of the change in the cysteine redox status. Further investigation is warranted to explore this switch as a drug-development target by focusing on small molecules that alter the redox state of *Plasmodium* SHMT to the stable and inactive oxidized state. This molecule could also serve as an adjuvant to augment the antimalarial activity of other drugs.

## 4. Conclusions   

Although SHMT has long been proposed to be a drug target for the development of anticancer agents and antibacterials, progress has been slower than with other enzymes of the folate-biosynthesis pathway. SHMT from various organisms is known to catalyze diverse reactions including THF-dependent and THF-independent reactions and can use different amino-acid substrates such as serine, glycine and β-phenylserine. Furthermore, *Plasmodium* and human SHMTs have recently been shown to catalyze both d- and l-isomers of serine in a THF-dependent manner (Pinthong *et al.*, 2014 ▶). Nonetheless, the underlying mechanism that controls enzyme activity is not obvious, as different lines of evidence support different mechanisms.

The crystal structures of complexes of *Pv*SHMT with l-Ser and d-Ser–(6*R*)-5FTHF revealed that the arrangement of the residues in the amino-acid binding pocket is restricted by the configuration of the bound amino-acid substrate, leading to different catalytic efficiencies towards each substrate isomer. Although both (6*R*)- and (6*S*)-5FTHF can bind *Pv*SHMT in the presence of l-Ser and d-Ser, different binding affinities and intermediates were observed depending on the stereoisomeric substrate pair between the amino acids and folates, suggesting that the occurrence of a specific stereoisomer pair has a role in controlling the enzyme activity. The plasticity and chemical space in the *Pv*SHMT active site are also factors in allowing the binding of different amino acids, as demonstrated previously (Sopitthummakhun *et al.*, 2009 ▶). These findings have several implications. For example, it is possible that SHMT follows different mechanisms for catalysis depending on the substrate present. Therefore, understanding the chemistry of PLP-dependent reactions and the structure of specific SHMTs is necessary. The knowledge gained may lead to other applications of this enzyme such as stereospecific biocatalysis (Gutierrez *et al.*, 2008 ▶; Kreuzman *et al.*, 1997 ▶). From a drug-discovery point of view, inhibitor design with stereoisomers should be performed cautiously as non-equivalent efficacy can be foreseen.

Based on the structure–function analysis of *Pv*SHMT, several types of antimalarials targeting SHMT can be designed. The first category comprises conventional inhibitors occupying the amino acid- or folate-binding site. For this type of inhibitor, the stereoisomer form should be taken into account in order to gain maximum efficacy, as demonstrated by biochemical and structural studies of d-Ser* versus* l-Ser and (6*S*)-5FTHF *versus* (6*R*)-5FTHF. Another consideration is the polarity of the compound. Compounds that mimic the polyglutamate moiety of the folate substrate would preferably inhibit the enzyme, as a large surface area of *Plasmodium* SHMT is highly positively charged. Owing to the different electrostatic surface potentials between bacterial and *Plasmodium* SHMTs, inhibitors can be specifically designed for malarial and bacterial SHMTs. Last but not least, inhibitors that irreversibly oxidize the disulfide bond of the cysteine redox switch in *Plasmodium* SHMT could constitute another family of promising antimalarial drugs.

## Supplementary Material

PDB reference: SHMT with l-serine Schiff base, 4pfn

PDB reference: with PLP Schiff base, 4pff

PDB reference: complex with d-serine and folinic acid, 4oyt

## Figures and Tables

**Table 1:** Data-collection and refinement statistics of *Pv*SHMT complexes

	PLP	PLPL-Ser	PLPD-Ser(6*R*)-5FTHF
Wavelength ()	1	1	1
Space group	*C*2	*C*2	*C*2
Molecules in asymmetric unit	3	3	3
Resolution ()	29.172.30 (2.382.30)	29.492.50 (2.592.50)	28.842.40 (2.492.40)
Unit-cell parameters (, )	*a* = 101.05, *b* = 58.33, *c* = 237.14, = 90.014	*a* = 100.40,* b* = 57.94, *c* = 235.92, = 90.014	*a* = 100.62,* b* = 58.12, *c* = 236.61, = 90.002
No. of measured reflections	130608	129610	120462
No. of unique reflections	58848	44633	51056
Multiplicity	2.4 (2.0)	3.0 (2.7)	2.5 (1.8)
Completeness (%)	94.6 (85.0)	94.1 (82.5)	94.6 (84.9)
*I*/(*I*)	27.2 (12.2)	38.73 (10.30)	20.4 (4.6)
*R*_merge_ † (%)	3.2 (6.0)	2.8 (10.6)	4.7 (17.2)
Wilson *B* factor (^2^)	34.9	44.3	47.7
Weight matrix in *REFMAC*5	0.05	0.04	0.06
FOM	0.826	0.804	0.804
*R* factor/*R*_free_ (%)	22.11/24.00	20.70/26.32	19.96/25.23
R.m.s. bond deviation ()	0.0072	0.0069	0.0083
R.m.s. angle deviation ()	1.2138	1.1374	1.3820
Ramachandran plot, residues in (%)			
Most favoured regions	91.2	90.7	90.6
Additional allowed regions	8.0	8.3	8.8
Generously allowed regions	0.8	0.8	0.6
Disallowed regions	0.0	0.2	0.0
PDB code	4pff	4pfn	4oyt

† *R*_merge_ = 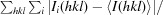 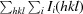, where *I_i_*(*hkl*) is the intensity of the *i*th measurement of an equivalent reflection with indices *hkl* and *I*(*hkl*) is the mean intensity of *I_i_*(*hkl*) for all *i* measurements.
